# Experimental Manipulation of Melanism Demonstrates the Plasticity of Preferred Temperature in an Agricultural Pest (*Phaulacridium vittatum*)

**DOI:** 10.1371/journal.pone.0080243

**Published:** 2013-11-06

**Authors:** Rebecca M. B. Harris, Peter McQuillan, Lesley Hughes

**Affiliations:** 1 Antarctic Climate Ecosystems Cooperative Research Centre, University of Tasmania, Hobart, Tasmania, Australia; 2 School of Geography and Environmental Studies, University of Tasmania, Hobart, Tasmania, Australia; 3 Department of Biological Sciences, Macquarie University, North Ryde, New South Wales, Australia; CNRS, Université de Bourgogne, France

## Abstract

Phenotypic plasticity is a key trait of successful pest species, and may increase the ability to cope with higher, more variable temperatures under climate change. We investigate the plasticity of preferred temperature in a widespread agricultural pest, the wingless grasshopper (*Phaulacridium vittatum*). Preferred temperature is a measure of thermoregulatory behaviour through habitat selection. It is influenced by melanism, which affects body temperature by determining the amount of radiation absorbed by the body. First we demonstrate that body temperature and preferred temperature in *P. vittatum* is influenced by melanism, by comparing the preferred temperature of the colour morphs in laboratory thermal gradients and field body temperatures in natural populations. We then test whether preferred temperature changes in response to changes in body temperature, by determining preferred temperature before and after manipulation of melanism by painting. When melanism was manipulated experimentally in live grasshoppers, preferred temperature changed to reflect the thermal qualities of the new colour. The preferred temperature of light grasshoppers increased after they were painted black, and decreased after being painted white. Similarly, dark individuals that were painted white behaved like a light individual, maintaining a lower body temperature. Preferred temperature in *P.vittatum* is a plastic thermoregulatory response to ambient temperature, mediated by the influence of melanism on body temperature.

## Introduction

The role of behavioural thermoregulation in ectotherms has recently been highlighted as a mechanism for coping with climate change [1]. Short-term, reactive behavioural responses enable ectotherms to regulate their body temperature under a range of environmental conditions [2]. However, another aspect of behavioural thermoregulation that may contribute to an ectotherm’s adaptive capacity is the phenotypic plasticity (hereafter referred to as plasticity) of preferred body temperature [3].

The preferred temperature, the ambient temperature to which an insect moves if given its choice of a temperature gradient [4], is a measure of thermoregulatory behaviour through habitat selection. As such it incorporates trade-offs between ecological thermal optima and physiological optima. The preferred temperature has been referred to as “one of the most potent factors influencing the distribution of insects and their movements” [5] . A range of performance measures of insects have been shown to be maximised at the preferred temperature, such as feeding rates and development in grasshoppers [6,7] and butterflies [8], reproductive optima in beetles [4], and brood survival and caste determination in ants [9,10].

Adaptation to changing conditions can occur through evolutionary adaptation (i.e., through natural selection acting on quantitative traits), but this requires many generations. In contrast, physiological and/or behavioural plasticity has the potential to alleviate or minimise climate change impacts in the short term [11]. Plasticity in preferred temperature may be able to partially or fully compensate for increasing temperatures in a changing climate, enabling activities such as feeding, mating and avoidance of predators to be maintained as environmental temperatures increase [3]. 

Plasticity is a key trait of many successful pest species. Plasticity in thermal tolerance has been shown to improve the fitness of invasive species such as the Mediterranean fruit fly (*Ceratitis capitata*) [12], the slug *Arion lusitanicus* [13], and species of collembolan springtails (*Pogonognathus* and *Isotomurus* species) [14,15]. Just as plasticity in thermal tolerance traits contributes to the current success of a species, it will also increase the potential to cope with changing climatic conditions in the future. Plasticity of preferred temperature will therefore be an important determinant of the likelihood that a species will become invasive, spread into new areas or reach outbreak population levels within current distributions [15,16]. 

The so-called wingless grasshopper, *Phaulacridium vittatum* (Sjöstedt), is an example of a species that might be expected to have high thermal flexibility and adaptability to changing conditions. It is a major agricultural pest in Australia, causing damage to grazing pasture and high value crops such as grapes, vegetables, fruit and tree nurseries [17]. It is a common, widely distributed generalist herbivore, able to exploit a wide range of habitats, from gardens and pastures to open woodland and forest margins [18]. It exhibits morphological polymorphisms in body size and melanism, and actively regulates its body temperature through behavioural means. We have shown elsewhere [19] that the degree of melanism (the occurrence of darker pigmentation in individuals), affects the thermal properties of the colour morphs in *P.vittatum*, with darker unstriped grasshoppers warming more rapidly and reaching a higher maximum body temperature than lighter grasshoppers. If melanism affects the thermal properties of grasshoppers, and thus the temperature experienced by an individual, it could also be expected to influence the preferred temperature.

Our primary aim was to test whether preferred temperature is a plastic trait in *P. vittatum*, responsive to changes in the temperature experienced by a grasshopper. First we established that body temperature and preferred temperature in *P. vittatum* is influenced by melanism, by comparing the preferred temperature of the colour morphs in laboratory thermal gradients and field body temperatures in natural populations. We then manipulated melanism using paint, to change the amount of radiation absorbed by the grasshoppers [20,21], and thus the body temperature experienced. Preferred temperature was measured before and after painting, to test whether preferred temperature can change in response to changes in body temperature.

## Methods

### Ethics statement

All grasshoppers were collected from unprotected public lands, for which no collecting permit is required under the Nature Conservation Act of 2002, or on private land where permission had been granted by the owner. Under the Tasmanian Animal Welfare Act (1993), ethics approval is not required for research involving non-protected invertebrates. No grasshopper was specifically killed for this study.

*P. vittatum* is a common acridid grasshopper, widely distributed in open habitats in the cool temperate areas of eastern and southern Australia (-23° 36’ to -43° 06’S latitude). Body size is variable, with females ranging in length from 12-20mm and males from 10-13mm [22]. There is also individual variability in the expression of striped patterning and functional wings, melanism and colour. Individuals can be light, through to dark brown and black, and rarely, green. Individuals can be striped, with two white longitudinal stripes on the dorsal surface, unstriped, or patterned, with very dark lateral surfaces on the pronotum and a light dorsal surface. Colour is set for an individual once it reaches the adult stage and a range of colour morphs is represented within populations [18].

Although melanism in *P. vittatum* represents a gradation in colour, with reflectance ranging from 2.49 to 5.65% [23], we have shown elsewhere that visual separation of the colour morphs into distinct categories can reliably be made, and that these categories relate to measurable differences in average reflectance in the UV and visible range [24]. In this study we separated unstriped grasshoppers into five colour morphs ranging from light to black, and striped grasshoppers into three colour morphs. Males were generally darker than females – no females were assigned to the darkest colour morph, and no males were assigned to the lightest. 

We performed the experiments only on adult wingless individuals (technically brachypterous), due to the difficulties of running behavioural experiments with winged grasshoppers, and the absence of several of the colour morphs in the winged form (e.g. the green and the very light patterned form). Winged individuals make up only a small proportion (<5%) of most populations, except those from high altitude populations along margins of relatively closed forest, where they are more abundant [25]. 

### Body temperatures of live grasshoppers in natural situations

Field body temperatures of grasshoppers were measured in March 2008 and April 2009 to assess whether a similar body temperature was maintained by all colour morphs in different habitats and altitudes throughout the species’ range. Body temperatures of live grasshoppers were measured from 15 populations ranging from close to the species’ northern limit to its southern limit in Tasmania. Seven Tasmanian sites (n = 179) and eight Australian mainland sites (n = 60) were included. These sites represent the range of altitudes (10m - 1500m above sea level (asl.)) and habitats (roadside verges, agricultural pasture and forest margins) in which *P. vittatum* is found along the east coast of Australia. Only wingless individuals are included in the analyses (n = 225).

Field body temperatures were measured using a Raytek handheld infra-red thermometer (accurate to ±2°C) from a distance of 1cm while the grasshopper was on the lid of a collecting jar with a hand cupped around it to eliminate wind. Temperature readings were generally taken within 20 seconds of capture. The “grab and stab” method was not used, as it was not possible to insert a thermocouple without the grasshopper becoming stressed and the body temperature being affected by the delay and the struggle involved. The core body temperature has been shown to be approximately equal to the surface temperature at steady state in small and medium sized grasshoppers, due to circulating haemolymph (Stower & Griffith 1966). Surface temperature at the point from which the grasshopper had jumped prior to being caught was also measured, so that the temperature excess (the difference between ambient and body temperature) could be calculated. Grasshoppers were not used if it was not possible to be sure where they had jumped from (e.g. under a grass clump or on bare ground), or if it took more than 1 minute to capture them. 

All grasshoppers were collected, and sex, colour morph, body size and time of day were recorded before release. Sample sizes of the colour morphs were: unstriped (lightest (n = 14), light (n = 60), medium (n = 54), dark (n = 38) and black (n = 18)), and striped (light (n = 16), medium (n = 50) and dark (n = 35)). We used the length of the right femur as a surrogate for body size rather than weight, because of unequal delays between collecting and returning to the laboratory. Femur length is closely correlated with body size and other size metrics in grasshoppers [26], and is more reliable than body length, which can change as specimens dry post mortem. Femur length was measured using handheld Vernier callipers (accurate to 0.02mm).

### Preferred temperature measured in thermal gradient

Live grasshoppers were collected by hand from six localities in central and southern Tasmania over a period of 5 days (2^nd^-7^th^ March, 2008). All grasshoppers were held in the laboratory under identical conditions for a minimum of 7 days prior to the experiment. They were housed at 23°C in plastic cups with sand in the base, moist cotton wool, and plantain (*Plantago major*) leaves provided for food. The presence of egg capsules was noted to account for reproductive status.

The preferred temperature was measured in laboratory thermal gradients constructed of a 185cm long by 15cm wide aluminium plate, with 10cm high sides. The plate was divided into 3 separate runways, each 5 cm wide, using plastic strips. The gradients were covered with a movable Perspex lid, with a centre hole through which the animals could be introduced into the runways and holes drilled every 2.5 cm to allow air circulation and insertion of thermocouples. A temperature gradient was established by placing one end of the plate on a hot plate and the other on ice. The surface temperature ranged from 7° to 60°C in both gradients, with a surface temperature of approximately 28°C at the entry point. These temperatures remained stable throughout each experiment and were consistent between runs. All runs occurred between 10am and 2pm. Fluorescent tubes in the ceiling provided a constant overhead light source.

Runs were performed for 15 minutes, which had been determined as sufficient time for the grasshoppers to come to rest in trial runs (n=20) that were left for 30 minutes. Surface temperature was measured at the point at which the grasshopper came to rest, using a fine wire copper-constantan thermocouple (Type T, diameter of individual wire components 0.2mm) connected to a Campbell Scientific CR10X Datalogger, via an AM 1632 Multiplexer and using the LoggerNet 3.2 program and a 107TP reference temperature probe. Individuals moved freely within the gradient. Each run included individuals belonging to different sexes and colour morphs, to ensure that there were no confounding effects of experimental run or gradient. Each individual was assessed for activity at the end of its run - any animal that did not jump to escape a probe was excluded. This generally occurred when an animal made an initial jump when released into the gradient which landed them at an extremely high temperature. It rarely occurred at low temperatures, as the grasshopper would slowly move out of the cold area. 

Individuals were weighed immediately after their first run, and assigned a colour code, from light to dark. There were 5 unstriped colour morphs (lightest, light, medium, dark, black) and 3 striped morphs (light, medium, dark), as above. 

### Manipulation of melanism

Melanism was manipulated by painting the thorax of light/dark individuals the opposite colour with water-based face paint. The grasshoppers were rested for one hour and then put through the thermal gradient a second time to determine preferred temperature. To assess any potential effect of the painting treatment, a subset of dark grasshoppers was painted black and light grasshoppers were painted white. A total of 78 grasshoppers was tested (39 males and 39 females). Individuals were put into the freezer at -20°C immediately after their last run for 15 minutes, enabling the femur length to be measured. After recovery, live grasshoppers were released. Numbers of each colour morph tested are shown in Table 1. Striped and winged grasshoppers were not included in this experiment. 

**Table 1:** Pair-wise comparisons of preferred temperature before and after painting.

**Colour morph**	**Painted black**	**Painted white**			
	Mean difference	Mean difference	F	P	df
**Lightest**	+4.55 (11)	-1.91 (9)	5.99	**0.03**	19
**Light**	+4.60 (15)	-4.25 (11)	11.74	**0.01**	25
**Medium**	+0.36 (4)	-5.75 (5)	2.24	0.18	8
**Dark**	+0.19 (8)	-10.92 (5)	9.30	**0.01**	12
**Black**	-1.17 (7)	-8.64 (3)	17.15	**0.01**	9

Mean difference is the difference in preferred temperature before and after painting. Comparisons significant at the 0.05 level are in bold.

#### Statistical analyses

We fitted a multiple regression model with randomisation of modified residuals [27], to test for a difference in field body temperature and/or temperature excess (the difference between ambient and body temperature) between colour codes within the striped and unstriped categories, while accounting for body size. We used this method because it enables the significance of the effect of any predictor to be tested, conditional on the effect of the remaining predictors, and is robust to violations of assumptions such as non-Normality of the distribution and inter-correlation between predictor variables [28]. Using this approach enabled data from the different populations to be combined, thus avoiding the problem of small sample sizes. Predictor variables included were colour code, femur length or weight, altitude, population (for field temperatures) and stripe category. Tests were carried out using MiniTab Version 16.1.0 [29] and the REGRESSRESRAN macro from [30]. All probabilities given refer to those for two-sided randomizations. 

For the laboratory thermal gradients, the Kruskal-Wallis test (non-parametric, 1 way ANOVA based on ranks) was used to test whether the colour morphs had significantly different preferred temperatures prior to painting. The Dwass-Steel-Critchlow-Fligner procedure , a non-parametric equivalent to the Tukey-Kramer HSD pair-wise test [31,32], was then used to determine where the differences lay. Significant differences in the preferred temperature of individuals before and after painting were tested using the Wilcoxon-Signed Rank test between matched pairs. Tests were done using JMP Version 10 [33].

## Results

### Field body temperatures in natural situations

**Figure 1:**
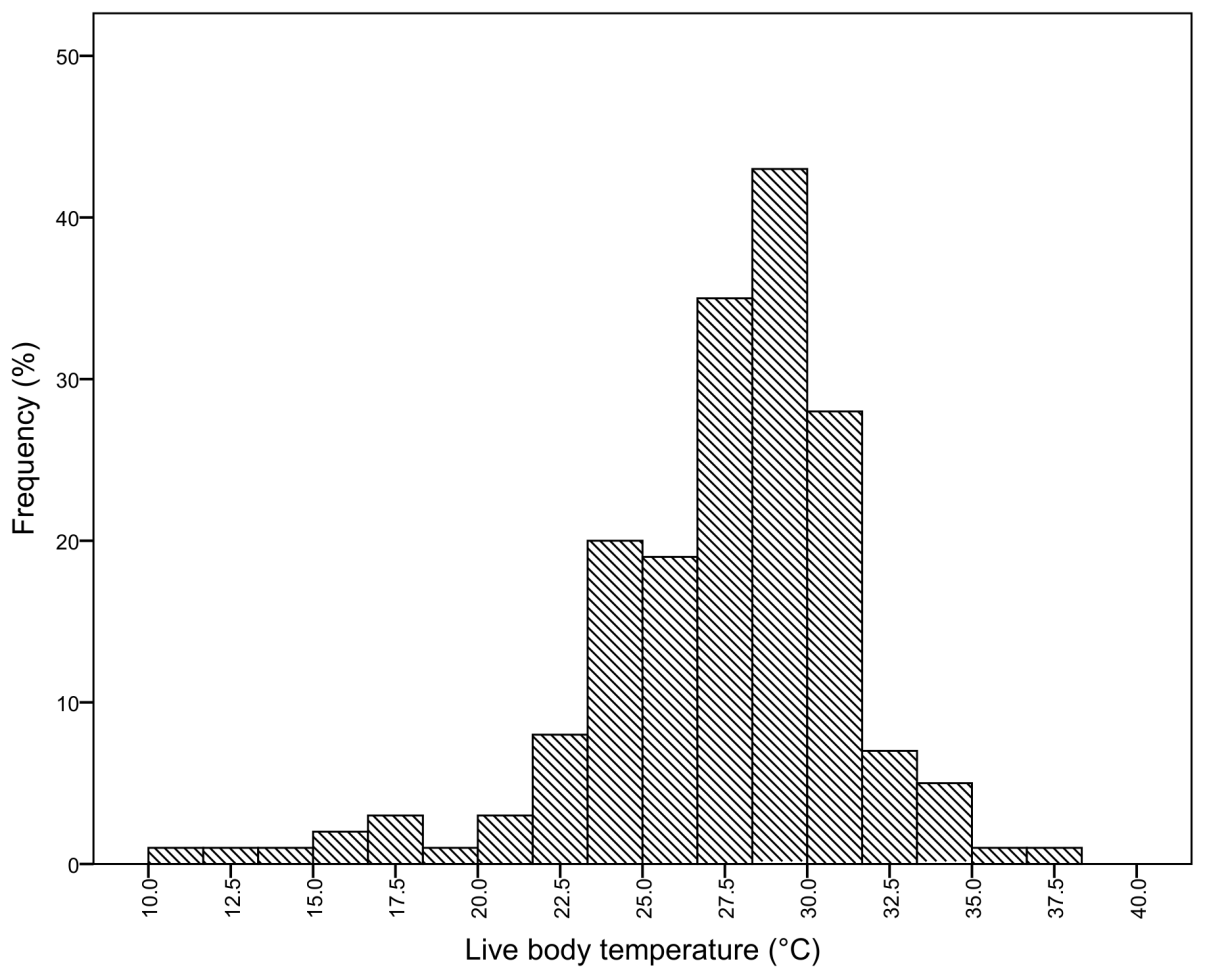
Frequency histogram of field body temperature in *Phaulacridium vittatum*.

The peak field body temperature range for the wingless grasshopper in natural situations was 27.5 - 30.0°C (Figure 1). The distribution was unimodal, with a fairly sharp avoidance of high temperatures above 35°C and low temperatures below 12°C. This value represents an overall estimate for all colour morphs collected from different habitats and altitudes throughout the range of *P.vittatum*.

Field body temperatures of males (n = 99) (26.91 ± 0.40°C) and females (n = 125) (27.15 ± 0.35°C) (F_1,222_ = 0.08; p =0.77) were not significantly different, and there was no difference in their temperature excess (F_1,222_ = 0.09; p = 0.77). They are therefore combined for subsequent analyses. The overall mean field body temperature was 27.04 ± 0.26°C.

There was no significant difference in temperature excess (F_1,222_ = 0.89; p > 0.05) or surface temperature (F_1,222_ = 0.89; p > 0.05) between sites sampled in the different seasons, so they are also combined for subsequent analyses. 

The field body temperatures of the colour morphs were significantly different (F_5,219_ = 4.20; p = 0.04) after body size, altitude and population was accounted for. This difference was driven by differences between the colour codes within the unstriped category. The darkest unstriped morph had a higher body temperature (28.8 ± 0.18°C, n = 11) than the lightest unstriped morph (26.4 ± 0.82°C, n = 23) (Tukey’s q = 2.60, p < 0.05). Body size was not a significant predictor of field body temperature (F_5,219_ = 3.54; p = 0.08). Temperature excess was not significantly different between different colours (F_5,219_ = 1.66; p = 0.19) or populations (F_5,219_ = 2.06; p = 0.14).

### Preferred temperatures

**Figure 2:**
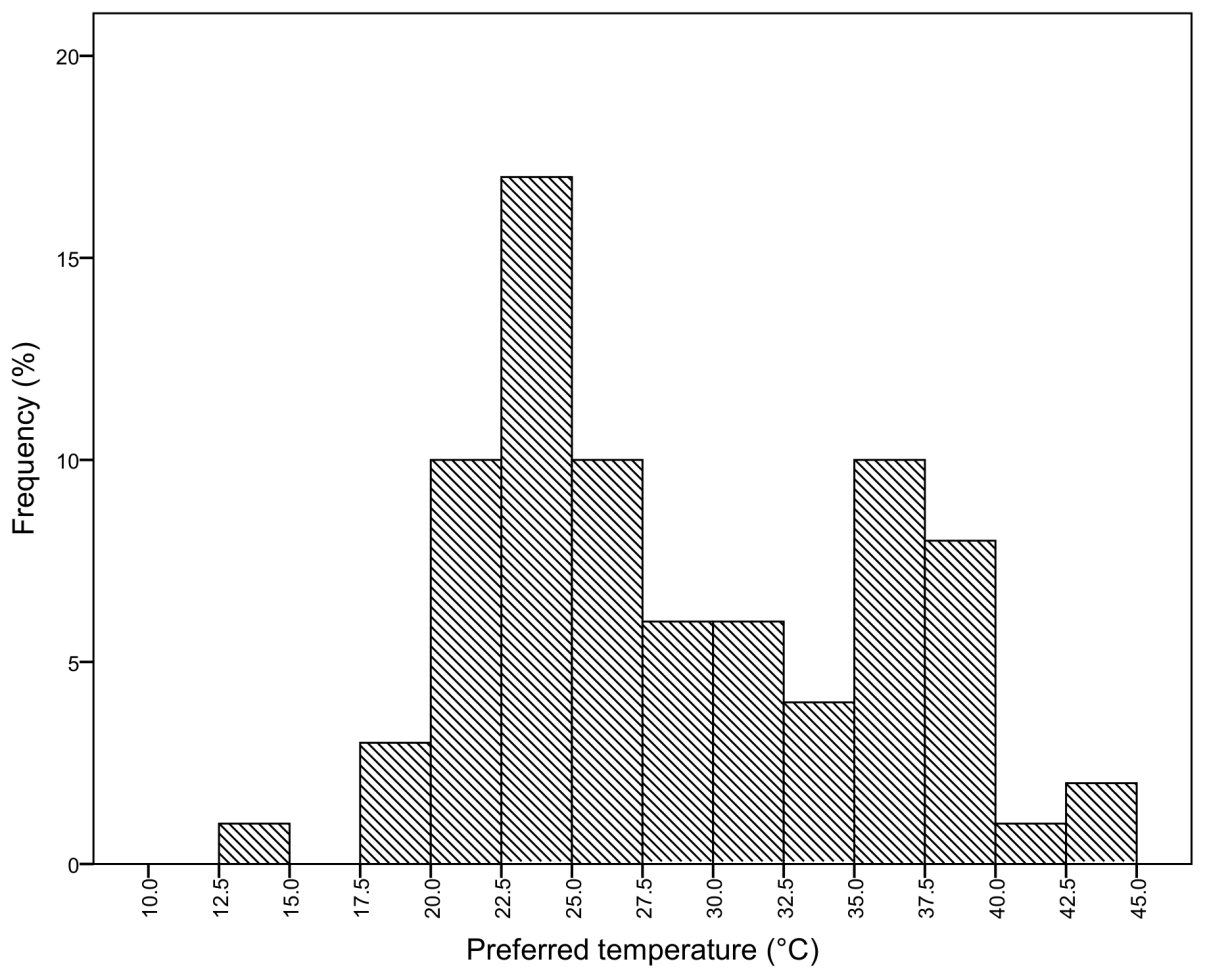
Frequency histogram of preferred temperature before painting.

The frequency distribution of the preferred temperature of grasshoppers before painting was bimodal, with a peak preferred temperature range of 22.5-25.0°C, and a high preferred temperature range of 35.0-37.5°C (Figure 2). However, diagnostic tests of normality (Q-Q Normal plot, Residuals plot) and homogeneity of variance (Levene’s homogeneity test) showed that the deviations were not extreme across all categories, or within colour or sex categories separately. 

**Figure 3:**
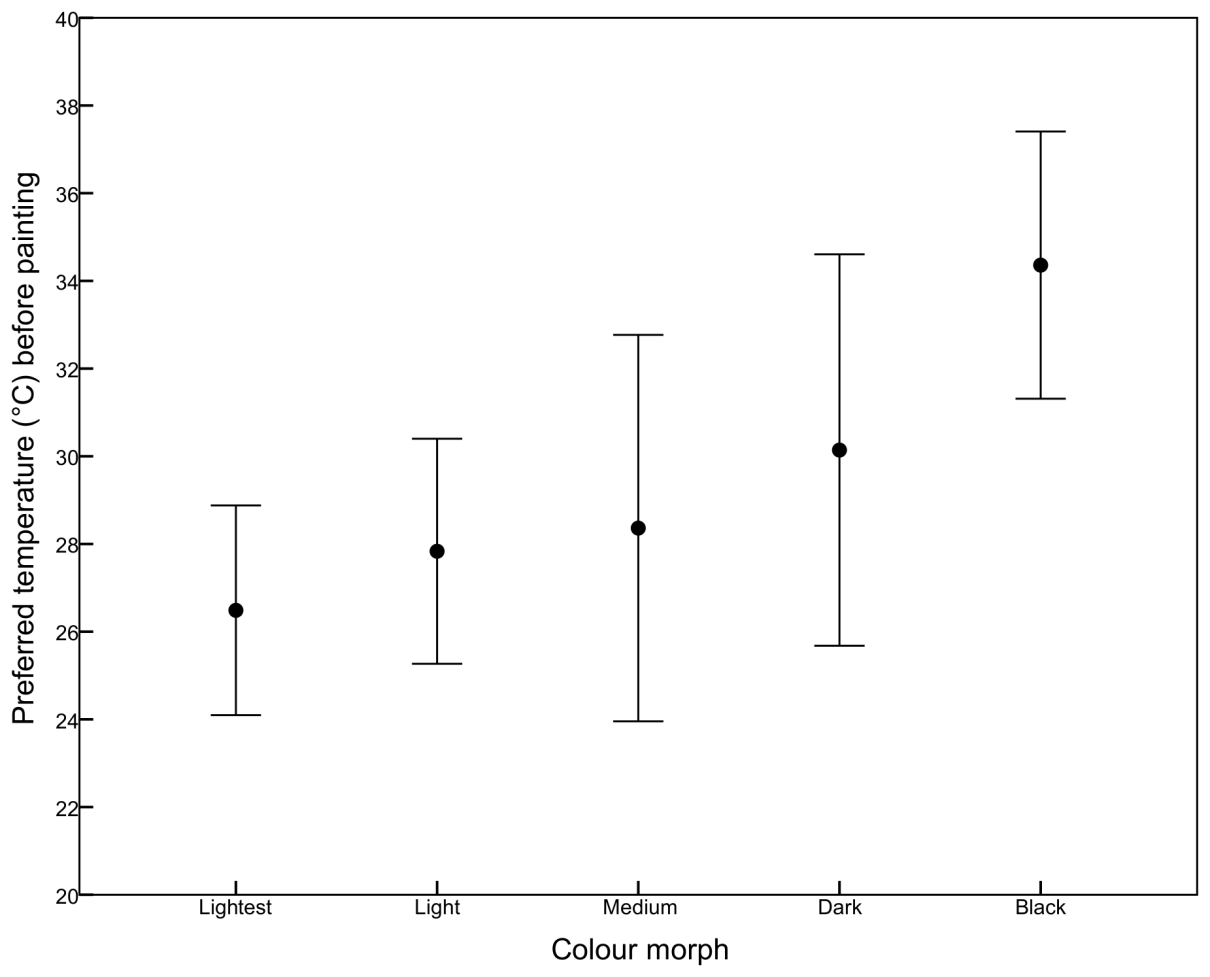
Preferred temperature (mean ± 2 se) of the colour morphs in the thermal gradient before painting.

Before painting, the preferred temperature of the colour morphs was significantly different (Kruskal-Wallis χ^2^ = 10.45, df = 4; p = 0.03). The black morph (n = 10) was significantly different from the lightest (n = 20) (Steel-Dwass z = 3.06, p = 0.02) and light morphs (n= 26) (Steel-Dwass z = 2.92, p = 0.03), with the black morph having a higher preferred temperature (34.4 ± 2.1°C) than the lightest (26.5 ± 1.4°C) and light morphs (27.8 ± 1.2°C). The intermediate colour morphs exhibited preferred temperatures between these extremes (Figure 3). 

There was a high degree of variability in the preferred temperature within morph categories, with a range in values of at least 5°C in the lightest, light and black morphs. The medium (n = 9) and dark (n = 13) colour categories exhibited greater variability, possibly reflecting the difficulty in accurately separating the intermediate morphs visually. 

### Manipulation of colour

**Figure 4:**
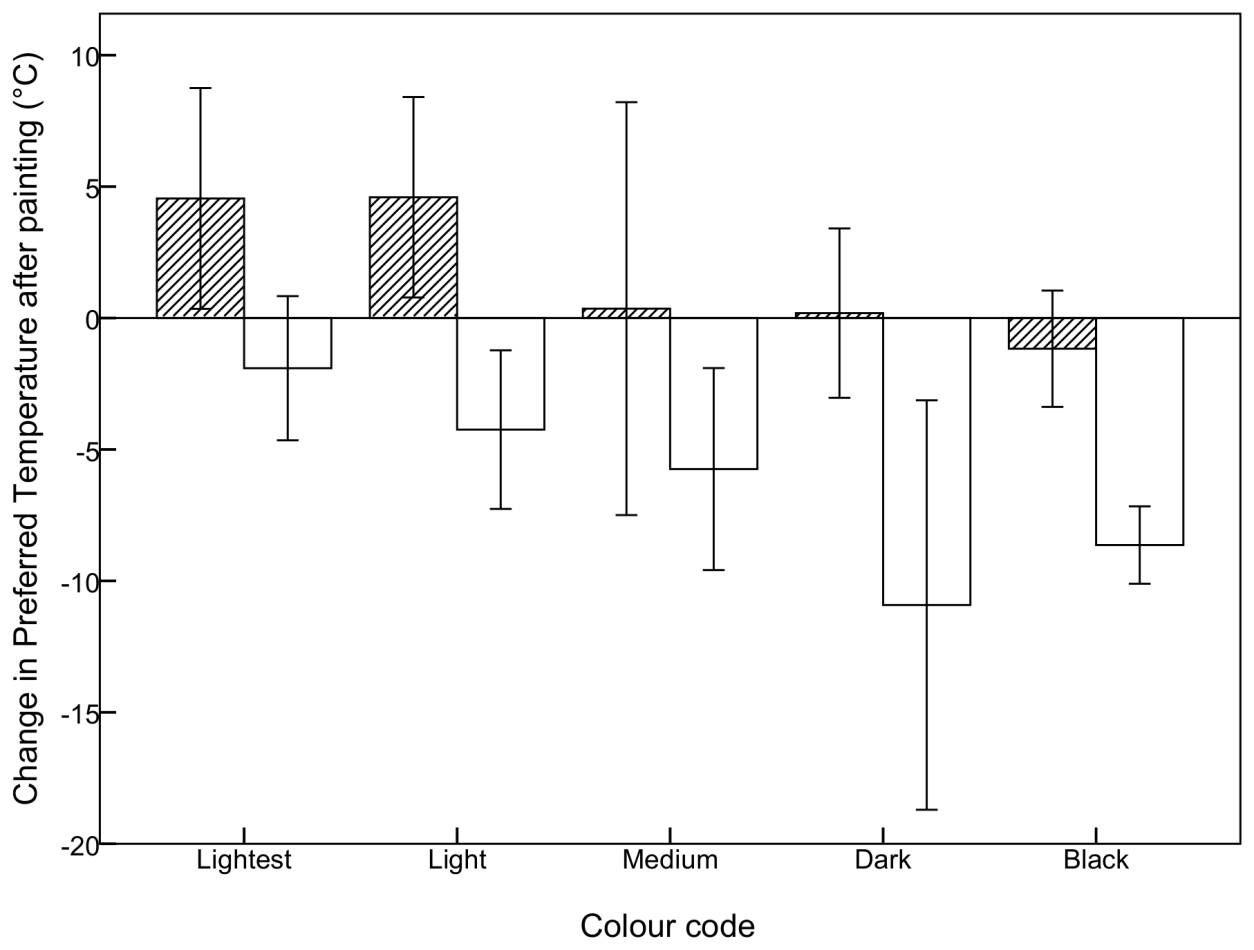
Change (± 2 se) in preferred temperature of *P.vittatum* after painting. Hatched bars are those individuals painted black, and white bars are those painted white.

The preferred temperature of individuals changed to reflect their new colour after painting (Figure 4). The preferred temperature of light grasshoppers increased after they were painted black, and decreased after being painted white. When dark and black grasshoppers were painted white, their preferred temperature decreased. Pair-wise comparisons for light grasshoppers painted black and all grasshoppers painted white, with the exception of the lightest morph, showed statistically significant differences after painting (Table 1). The white paint used here was substantially whiter than the lightest naturally occurring colour in the wingless grasshopper, so a decrease in preferred temperature after being painted white supports expectations. Painting medium, dark and black grasshoppers with black paint had little effect on the preferred temperature, as expected, because there was little difference in hue between the painted black and the natural black. 

## Discussion

The adaptive capacity of an organism is determined by its ecological, genotypic and phenotypic plasticity [34], as well as its historical biogeography and genetic diversity [11]. Interactions between these traits may also be important, as we have highlighted here. In the wingless grasshopper, it is the interaction between behavioural thermoregulation, an example of ecological plasticity, and melanism that determines the adaptive capacity in preferred temperature.

Although behavioural thermoregulation may provide a short-term buffer against the impacts of climate change [1], in the long-term it may inhibit adaptation to climate change since it reduces exposure to elevated temperatures and the intensity of selection on other traits [35]. However, plasticity in preferred temperature may provide a mechanism for coping with climate change in the longer term. 

The preferred temperature of *P.vittatum* is influenced by the degree of melanism, as shown both in laboratory thermal gradients and field populations. The preferred temperature of the darkest colour morph was, on average, approximately 5°C higher than the lightest morphs in the thermal gradient before colour was manipulated (34.4 ± 2.1°C, compared to 26.49 ± 1.4°C). The darkest morph also maintained a higher body temperature in natural situations, although the difference was not as marked (28.8 ± 0.18°C, compared to 26.34± 0.82°C). Intermediate morphs showed a gradation in both field body temperatures and preferred temperatures that related to their degree of melanism. 

The field body temperatures in natural situations were slightly higher than the preferred temperatures measured in the thermal gradient. The difference may be an artefact of the different methodology used to measure the temperature. A hand-held infra-red thermometer was used to measure field body temperatures in natural situations, while thermocouples were used to measure temperature in the laboratory thermal gradient. Alternatively, the difference may reflect the compromise between physiology and ecology in natural situations (Huey & Stevenson 1979). The average field body temperature reflects the combination of all fitness characteristics being maximised, all of which may have slightly different thermal requirements (Stevenson, Peterson & Tsuji 1985). For example, one temperature may be chosen to maximise development, then another to maximise feeding and another for reproduction. In comparison, in thermal gradients only the effect of temperature is being measured (Thiele 1977). The grasshoppers are selecting temperatures without being constrained by factors such as predation, low light levels or unsuitable transitional microhabitats that they would commonly be exposed to under natural conditions (O'Neill & Rolston 2007).

By manipulating melanism with paint, we have shown that the preferred temperature is a plastic thermoregulatory response to changes in the thermal balance. Melanism affects body temperature by changing the amount of radiation absorbed, causing darker grasshoppers to warm more rapidly and reach a higher maximum temperature than lighter grasshoppers [19]. When melanism was changed, the grasshopper behaviour changed to reflect the thermodynamic properties of the new colour. The preferred temperature of light grasshoppers increased after they were painted black, and decreased after being painted white. Similarly, dark individuals that were painted white behaved like light individuals, maintaining a lower body temperature. The body temperature maintained by a grasshopper is therefore a plastic response to ambient temperature, rather than genetically determined. 

It is possible, however, that while these changes are initially behaviourally and physiologically mediated within an individual, the degree of plasticity may have an additive genetic component. If plastic responses are favoured by selection via increasing survival and reproduction, these responses may become fixed in populations over time [11].

In common with many other pest species, *P. vittatum* has a broad and flexible thermal tolerance range. There was a high degree of variability in the preferred temperature within morph categories, within populations, and even within individuals. The existence of different colour morphs extends the collective range in preferred temperature of the species. Plasticity in preferred temperature then further enhances the ability of this species to respond in the short term to increasingly high and variable temperatures. This has the potential to facilitate its spread into new areas, change inter-specific interactions and increase the scale and frequency of outbreaks, caused by changes in population growth rates or increases in the number of generations per year [16,36]. 

Although we have focused on one species of insect, the results are illustrative of the potential capacity for other small ectotherms with similar characteristics to cope with temperature change. Conversely, many species do not possess these characteristics, and these species are likely to be more vulnerable to negative impacts in a rapidly changing climate.

## Conclusions

We have demonstrated that thermoregulatory behaviour through habitat selection in *P.vittatum* is a plastic response to changes in the thermal balance driven by melanism. Experimental manipulation of melanism led to changes in preferred temperature, with behaviour changing to reflect the thermo-dynamic properties of the new colour. 
